# An up-to-date catalog of available urinary biomarkers for the surveillance of non-muscle invasive bladder cancer

**DOI:** 10.1007/s00345-018-2380-x

**Published:** 2018-06-21

**Authors:** Francesco Soria, Michael J. Droller, Yair Lotan, Paolo Gontero, David D’Andrea, Kilian M. Gust, Morgan Rouprêt, Marek Babjuk, Joan Palou, Shahrokh F. Shariat

**Affiliations:** 1 Department of Urology and Comprehensive Cancer Center, Vienna General Hospital, Medical University of Vienna, Waehringer Guertel 18-20, 1090 Vienna, Austria; 2 Division of Urology, Department of Surgical Sciences, San Giovanni Battista Hospital, University of Studies of Torino, Turin, Italy; 3 Department of Urology, The Mount Sinai Medical Center, New York, USA; 4 Department of Urology, University of Texas Southwestern Medical Center, Dallas, USA; 5 Department of Urology, Hôpital Pitié-Salpétrière, Assistance Publique—Hôpitaux de Paris Sorbonne Université, Paris, France; 6 Department of Urology, Hospital Motol, Second Faculty of Medicine, Charles University, Prague, Czech Republic; 7 Department of Urology, Fundació Puigvert, Universidad Autónoma de Barcelona, Barcelona, Spain; 8 Department of Urology, Weill Cornell Medical College, New York, USA; 9 Karl Landsteiner Institute of Urology and Andrology, Vienna, Austria

**Keywords:** Urinary biomarker, Test, Follow-up, Surveillance, Non-muscle invasive bladder cancer, Recurrence

## Abstract

**Objectives:**

With the advent of novel genomic and transcriptomic technologies, new urinary biomarkers have been identified and tested for bladder cancer (BCa) surveillance. To summarize the current status of urinary biomarkers for the detection of recurrence and/or progression in the follow-up of non-muscle invasive BCa patients, and to assess the value of urinary biomarkers in predicting response to intravesical Bacillus Calmette–Guerin (BCG) therapy.

**Methods and materials:**

A medline/pubmed© literature search was performed. The performance of commercially available and investigational biomarkers has been reviewed. End points were cancer detection (recurrence), cancer progression, and response to BCG therapy.

**Results:**

The performance requirements for biomarkers are variable according to the clinical scenario. The clinical role of urinary biomarkers in the follow-up of non-muscle invasive BCa patients remains undefined. The FDA-approved tests provide unsatisfactory sensitivity and specificity levels and their use is limited. Fluorescence in situ hybridization (FISH) has been shown to be useful in specific scenarios, mostly as a reflex test and in the setting of equivocal urinary cytology. FISH and immunocytology could conceivably be used to assess BCG response. Recently developed biomarkers have shown promising results; upcoming large trials will test their utility in specific clinical scenarios in a manner similar to a phased drug development strategy.

**Conclusions:**

Current commercially available urinary biomarker-based tests are not sufficiently validated to be widely used in clinical practice. Several novel biomarkers are currently under investigation. Prospective multicenter analyses will be needed to establish their clinical relevance and value.

## Introduction

Bladder cancer (BCa) has the highest lifetime cost per patient of all cancers [1]. This is mainly due to the high recurrence rate and the consequent need for close, long-term follow-up based on the patient’s risk profile. Follow-up generally consists of regular surveillance cystoscopy and voided urine cytology with periodic upper urinary tract imaging [3]. The intensity and invasiveness of these tests can lead to morbidity, compromise in patients’ quality of life, and financial burden [4].

Use of urinary biomarkers could obviate these and potentially either detect disease recurrence before it becomes visually apparent or exclude its presence. Unfortunately, the most used urinary test, cytology, suffers from insufficient reproducibility and robustness to suffice for utility in various clinical scenarios such as surveillance of the most common type of BCa (low-grade NMIBC) [5, 6]. Recent multicenter studies have also shown a lower sensitivity of cytology for high-grade cancer compared to historical data [7, 8]. Urinary biomarkers in the surveillance setting have three aims: to reduce the frequency of invasive testing while still detecting early disease recurrence (Rec), to exclude the presence of recurrent disease and to detect progression (Prog) in non-muscle invasive bladder cancer (NMIBC) patients and predicting response to therapies [9].

Several urinary biomarkers have been investigated according to these objectives. Six of them have been approved by the FDA in the follow-up of NMIBC patients. However, the trials designed to determine their accuracy were not sufficient to determine their clinical utility. Low positive predictive values due to insufficiently robust specificity have undermined the ability to use them in clinical practice in most settings. Actually, despite urologists can be considered early adopters of new technologies (in this case, of new markers), the ability to maintain innovations is influenced from performances, impact on decision-making and costs [10]. As such, the use of urinary markers in the surveillance of NMIBC remains limited. To overcome these limitations, a new generation of biomarkers has been developed and is currently under investigation, with initial results reported as “promising”.

We sought to review the current literature on urinary biomarkers used in the surveillance of NMIBC patients to discuss recent findings through a clinical lens and to create an up-to-date reference point.

## Methods and materials

A medline/pubmed© literature search was performed with different combinations of the terms “urinary biomarker”, “bladder cancer”, “superficial bladder cancer”, “non-muscle invasive bladder cancer”, “surveillance”, “follow-up”, “performance”, and “BCG response”. No time period restriction was set. Original articles, reviews and editorials were selected based on their clinical relevance. Cited references from selected articles were analyzed to find and include significant papers missed by our search. The performance of commercially available and investigational biomarkers was reviewed. End points were cancer detection (Rec), cancer Prog, and response to BCG therapy.

## Performance criteria desired and urinary markers evaluation according to quality of research: general considerations

The performance of biomarkers depends upon their sensitivity, specificity, positive predictive value (PPV) and negative predictive value (NPV). A marker’s predictive value is influenced by the prevalence of the disease in the population. In tumors, the value of urinary markers according to their sensitivity and specificity varies according to specific clinical scenarios and can influence their predictive value. This should be taken into account during the evaluation process.

Furthermore, an evaluation of biomarkers should not overlook their “clinical utility rating”. Since the development phases for biomarkers discovery and application are not dissimilar to those of therapeutic drugs, a phased approach evaluation scale has been proposed [11, 12]. This consists of a sequence of consecutive phases that can be summarized as: (1) preclinical exploratory studies; (2) assay development (phase 0); (3) small retrospective series (phase I); (4) independent validation of the accuracy in larger series (phase II); (5) external validation in retrospective/prospective multicenter series and prospective clinical trials (phase III) and (6) post-approval reports (phase IV) [13]. Based on this scale, it is possible to evaluate the current status of biomarker development processes to understand their role in clinical practice.

To date, five urinary tests (NMP22 test kit, NMP22 BladderChek Test, BTA TRAK, BTA stat, and UroVysion) have been approved by the US Food and Drug Administration (FDA) in the detection and surveillance setting, while the uCyt + test is only approved in the follow-up of NMIBC patients. However, none of them have been broadly implemented in clinical practice. Furthermore, none are recommended in international guidelines [3, 4, 14]. Numerous novel biomarkers have been tested and are currently under development (phase I–II–III). Thus far, they have not been approved by the FDA or other regulative agencies. However, one must also note that many markers are commercially available with no goal to become FDA-approved. There is no requirement to seek FDA approval for use in the US. While it can help with reimbursement from some insurance companies, it does limit the ability to modify the marker by either using additional markers in a panel or changing cut-points. If the marker is already FDA-approved, then all the studies need to be repeated if the marker is modified even in the slightest. This is a roadblock for refining some of the already approved markers.

## Urinary biomarkers for the surveillance of bladder cancer patients

### Performance criteria desired

The performance criteria for urine markers highly depend on the clinical question to be answered. As with any test (laboratory, imaging, etc.), the result of the test depends heavily on the clinical setting. Since the goals for a marker may be different in a low- vs. high-risk patient or in first vs. fifth year of surveillance, the likelihood of cancer (prevalence) and projected use of the urine marker should impact the type of marker and designed performance characteristics.

For example, if the goal is to avoid a cystoscopy when a marker is negative, the marker has to have a very high NPV. This is especially important in high-grade disease, where a missed cancer due to a negative marker test could have a major impact on disease progression. On the other side, markers with a high sensitivity are needed if the tests are used in a setting where an improved detection is needed such as in patients at 3 months after initial TURBT or in those with high-risk disease. There is broad recognition from studies on enhanced cystoscopy that white-light cystoscopy alone can miss disease, especially carcinoma in situ (CIS) so that a marker can help to identify missed cancer. Most marker studies were not designed to biopsy patients with normal cystoscopy, but an abnormal biomarker. As such, it is not clear if an abnormal marker in the setting of a normal white-light cystoscopy implies the presence of cancer. Patients with a positive cytology are usually taken to the operating room for a biopsy and selective upper tract cytology, but that is not the case for positive markers. The concept of anticipatory positives is based on the concern that a marker can detect disease before it is visualized, but it is still not clear if this is clinically useful information [15].

Finally, predicting response to intravesical therapies such as BCG requires different characteristics than simply an improved detection and the role of urinary biomarkers in this setting is unclear so far.

### Urinary markers rated according to quality of research—according to phased approach

The performances of phase III–IV and phase II–III markers are summarized in Table 1.

**Table 1:** Summary of phase II–IV and phase II–III marker’s performances and their actual role in clinical practice for the surveillance of non-muscle invasive bladder cancer patients

Marker	Sensitivity (range, %)	Specificity (range, %)	PPV (range, %)	NPV (range, %)	Actual role in clinical practice^a^
NMP22 BladderChek	11–85.7	77–100	18.2–100	61.9–93.9	–
NMP22	24–81	49–100	31–100	60–91	–
BTA STAT	40–72	29–96	40–88	38–76.9	–
BTA Trak	50–62	68–87	45.4	88.4	–
ImmunoCyt	50–85	62–86	26–72	81–93	Could be used as reflex test in case of unsuspicious cystoscopy and equivocal/atypical cytology
UroVysion	13–100	63–100	21–83	67.9–100	Could be used as reflex test in case of unsuspicious cystoscopy and equivocal/atypical cytology
Cxbladder monitor	91–93	–	–	96–97	–
Bladder cancer (UBC) test	12–80	77.3–97	65.5–71.4	73.9–76.6	–

*PPV* positive predictive value, *NPV* negative predictive value

^a^Based on international guidelines’ recommendations

#### Phase III–IV markers

##### Nuclear matrix protein 22 (NMP22)

Nuclear matrix proteins are a structural part of the cell nucleus and provide support for the nuclear shape. A member of this family, NMP22, has been found to be elevated in malignant urothelial cells compared to normal urothelium, and is released in the urine as the result of apoptosis. Two assays have been developed to detect the presence of NMPs in urine. The first, NMP22BC is a quantitative Elisa test; the NMP22 BladderChek is a qualitative point-of-care (POC) test [16].

Independent of the type of test (qualitative or quantitative), a pooled analysis of seven studies with 4384 patients with previously treated BCa showed a sensitivity and specificity across all studies of 69% (range 50–85%) and 81% (range 46–93%), respectively [17].

Fourteen studies reported the performance of NMP22 BladderChek while 15 reported on NMP22BC (Table 2). According to a phased approach, NMP22 tests reached phase III (independent confirmation studies). Recently, four marker-comparison studies have been published, reporting low sensitivity rates of the qualitative test in detecting Rec (11–58%) [7, 18–20]. In contrast, the quantitative NMP22BC test is measured on a continuous scale and then transformed into a dichotomous variable by choosing a cut-off threshold (estimated at 10 ng/mL). From a biologic point of view, the use of a cut-off does not make much sense in the context of the continuity that the appearance of this marker represents, and therefore, generally leads to lower overall accuracy. Indeed, NMP22 BladderChek had a very poor sensitivity level for detecting Rec (26%), which only slightly outperformed that obtained with NMP22BC in the same population [7]. However, in a decision curve analysis, NMP22 was associated with oncological outcomes; it has been suggested that this test could be useful in decision-making between an immediate and delayed cystoscopy, depending on a clinician’s threshold to perform a surveillance cystoscopy [21].

**Table 2:** List of studies investigating the performances of NMP22 tests in surveillance setting in non-muscle invasive bladder cancer patients

Marker	Author (year) reference	Study design	No. of patients	Sensitivity (%)	Specificity (%)	Other end points
NMP22 BladderChek	Aguilera Tubet (2005) [65]	Cohort/marker comparison	88	28	93.55	–
	Grossman (2006) [66]	Cohort prospective	668	49.5	87.3	NPV 90.5%
	Kumar (2006) [67]	Cohort	131	85	77	PPV 67.2%, NPV 90.4%
	Gonzalo Rodriguez (2008) [68]	Cohort	109	25	91.1	PPV 18.2%, NPV 93.9%
	Gupta (2009) [69]	Cohort prospective	145	85.7	77.5	PPV 70.6%, NPV 89.6%. association with rec
	Kundal (2010) [70]	Cohort	115	81.3	92	–
	Choi (2010) [71]	Cohort	262	72.7	91.7	–
	Hwang (2011) [72]	Cohort	597	22.6	97.9	–
	Coskuner (2012) [73]	Cohort	95	44.4	98.4	PPV 80%, NPV 92.6%
	Önal (2015) [74]	Cohort/case control	65	85.4	76.5	–
	Yafi (2015) [17]	Marker-comparison prospective	109	58	85	–
	Bell (2016) [18]	Marker-comparison prospective	91	–	–	Association with RFS and PFS
	Lotan (2017) [7]	Marker-comparison prospective	803	11	–	NPV 86%
	Pichler (2017) [19]	Marker-comparison prospective	75	12.9	100	PPV 100%, NPV 61.9%
NMP22	Serretta (1998) [75]	Cohort	137	71	61	PPV 44.7%, NPV 83%
	Witjes (1998) [76]	Cohort	50	75	82	PPV 56%, NPV 91%
	Serretta (2000) [77]	Marker-comparison prospective	179	74	55	PPV 42.2%, NPV 83%
	Chahal (2001) [78]	Cohort	115	24	92	PPV 33%, NPV 87%
	Giannopoulos (2001) [79]	Marker-comparison prospective	95	56	–	–
	Shariat (2004) [80]	Cohort/case control	302/42	66 (cut-off 6.5 U/ml) 54 (cut-off 10 U/ml)	73 (cut-off 6.5 U/ml) 85 (cut-off 10 U/ml)	Prediction of BCa (OR 1.5)
	Aguilera Tubet (2005) [65]	Cohort/marker comparison	88	35	80.3	–
	Lahme (2005) [81]	Cohort/case control prospective	164/64	62	65.9	–
	Kibar (2006) [82]	Cohort/case control marker comparison	60/30	73	89.7	–
	Raina (2008) [83]	Cohort (atypical cytology)	71	81	89.3	PPV 92%, NPV 76%
	Mansoor (2008) [84]	Cohort	94	45	100	PPV 100%, NPV 87%
	Horstmann (2009) [85]	Cohort	221	68	49	PPV 57%, NPV 60%
	Shariat (2011) [20]	Cohort (negative cytology)	2222	–	–	Association with Rec and Prog
	Doğan (2013) [86]	Cohort	49	33	76	PPV 31%, NPV 78%
	Lotan (2017) [7]	Marker-comparison prospective	803	26	–	NPV 86%

*PPV* positive predictive value, *NPV* negative predictive value, *Rec* recurrence, *Prog* progression, *RFS* recurrence-free survival, *PFS* progression-free survival

The predictive value of the tests has also been evaluated with discordant results. Only few studies have found an association between NMP22 and future events such as Rec and Prog [19, 21, 22].

##### Bladder tumor antigen (BTA)

Bladder tumor antigen (BTA) tests detect the presence of basement membrane factors in the urine, which are released from tumor cells during stromal invasion. The BTA test exists as two assays: a quantitative Elisa-based assay (BTA TRAK) and a qualitative POC test (BTA stat).

Only a small number of studies have investigated the role of BTA tests in the follow-up of patients with NMIBC (Table 3). The majority of these are phase II–III studies, although no study assessed the value of these tests in a decision-making process. Sensitivity and specificity have varied from 54 to 61% and from 74 to 86%, respectively [23–25]. These measures are affected by the presence of concomitant conditions such as stones and “benign” genitourinary diseases [26]. Moreover, BTA tests have not been associated with future events such as Rec and Prog. Bell et al. compared the performances of different biomarkers and demonstrated that neither BTA STAT nor BTA Trak was associated with recurrence-free (RFS) or progression-free survival (PFS) [19]. Due to the low sensitivity and high rates of false-positive results, use of BTA tests cannot be recommended for use in clinical routine.

**Table 3:** List of studies investigating the performances of BTA tests in surveillance setting in patients with non-muscle invasive bladder cancer

Marker	Author (year) reference	Study design	No. of patients	Sensitivity (%)	Specificity (%)	Other end points
BTA STAT	Sarosdy (1995) [25]	Cohort prospective/case–control (separate)	499/564	40	96	–
	Ianari (1997) [87]	Cohort	75	54	91	–
	Leyh (1997) [88]	Cohort prospective	164	54	92	–
	Hargreave (1997) [89]	Cohort prospective	272	58	86	–
	Sarosdy (1997) [90]	Cohort retrospective/case–control (separate)	220/550	67	29-95	–
	Serretta (2000) [77]	Marker-comparison retrospective	179	57	62	PPV 40%, NPV 76.9%
	Giannopoulos (2001)	Marker-comparison prospective	95	72	–	–
	Sarosdy (2002) [91]	Cohort/case–control (separate)	438	50	–	–
	Lokeshwar (2002) [24]	Cohort prospective	26	61	74	PPV 88%, NPV 38%
	Raitanen (2008)	Cohort prospective	501	56	86	–
	Yafi (2015) [17]	Marker-comparison prospective	109	61	78	–
	Bell (2016) [18]	Marker-comparison prospective	91	–	–	No association with RFS or PFS
BTA Trak	Gutierrez Banoz (1999) [92]	Cohort	122	61	87	–
	Serretta (2000)	Marker-comparison retrospective	179	62	79	PPV 45.4%, NPV 88.4%
	Sarosdy (2002) [91]	Cohort/case–control (separate)	438	50	–	–
	Fernandez Gomez (2002) [93]	Cohort	Unknown (700 samples)	62	68	–
	Babjuk (2008) [22]	Cohort prospective	88	54	84	–
	Bell (2016) [18]	Marker-comparison prospective	91	–	–	No association with RFS or PFS

*PPV* positive predictive value, *NPV* negative predictive value, *RFS* recurrence-free survival, *PFS* progression-free survival

##### ImmunoCyt/uCyt+

The ImmunoCyt test is an immunocytological assay based on the microscopic detection of tumor cell antigens by immunofluorescence. The ImmunoCyt test measures the immunocytological expression of sulfated mucin-glycoproteins and glycosylated forms of the carcinoembryonic antigen in urine. In patients presenting with painless hematuria, immunocytology improves the diagnostic accuracy of standard predictive models by a clinically and statistically significant margin [27].

Sensitivity and NPV rates of ImmunoCyt vary between 62–85% and 74–93%, respectively (Table 4). In 942 patients, Mian et al. found that the sensitivity increased with pathologic grade (79.3% for G1, 84.1% for G2 and 92.1% for G3 tumors) [28]. In a prospective study, Bell et al. found no association of ImmunoCyt with either RFS or PFS [19]. The test is performed under microscopy and requires trained and experienced pathologists. Interobserver variability has been found to be a major drawback. The limited evidence of its benefit led to infrequent usage of the test [16].

**Table 4:** List of studies investigating the performances of ImmunoCyt test in surveillance setting in non-muscle invasive bladder cancer patients

Author (year) reference	Study design	No. of patients	Sensitivity (%)	Specificity (%)	Other end points
Vriesema (2001) [94]	Cohort	86	50	73	PPV 39%, NPV 81%
Pfister (2003) [95]	Cohort	458	70	82	–
Tetu (2005) [96]	Cohort	904	74	62	PPV 26%, NPV 93%
Messing (2005) [97]	Cohort	341	81	75	–
Lodde (2006) [98]	Cohort	195	84	86	PPV 63%, NPV 92%
Mian (2006) [27]	Marker-guided prospective	942	85	72	–
Sullivan (2009) [99]	Cohort	100	76	63	PPV 43%, NPV 88%
Horstmann (2009) [85]	Cohort	221	73	62	PPV 72%, NPV 74%
Yafi (2015) [17]	Marker-comparison prospective	109	62	79	–
Bell (2016) [18]	Marker-comparison prospective	91	–	–	No association with RFS or PFS

*PPV* positive predictive value, *NPV* negative predictive value, *RFS* recurrence-free survival, *PFS* progression-free survival

##### UroVysion (FISH)

UroVysion is a multicolor fluorescence in situ hybridization (FISH) containing probes to the centromeres of chromosomes 3, 7, 17 and the 9p21 locus (P16 tumor-suppressor gene). Sensitivity and specificity rates in detecting Rec vary from 13 to 94% and from 63 to 100%, respectively (Table 5).

**Table 5:** List of studies investigating the performances of UroVysion test in surveillance setting in non-muscle invasive bladder cancer patients

Author (year) reference	Study design	No. of patients	Sensitivity (%)	Specificity (%)	Other end points
Sarosdy (2002) [91]	Cohort/case–control (separate)	438	71	94	–
Varella-Garcia (2004) [100]	Cohort	19	87	100	–
Pycha (2004) [101]	Cohort	49	35	86	–
Kipp (2005) [102]	Cohort prospective	37	48	100	–
Bergmann (2007) [103]	Cohort	41	77	93	PPV 83%, NPV 90%
Moonen (2007) [104]	Cohort	105	39	90	–
Yoder (2007) [105]	Cohort, reflex (negative cytology)	249	73	87	–
Gudjónsson (2008) [106]	Cohort	159	30	95	–
Sullivan (2009) [99]	Cohort	100	13	90	PPV 33%, NPV 72%
Horstmann (2009) [85]	Cohort	221	76	63	PPV 68%, NPV 71%
Karnwal (2010) [107]	Cohort	59	63	65	–
Schlomer (2010) [29]	Cohort (atypical cytology and negative/equivocal cystoscopy)	73	100	67	PPV 21%, NPV 100%
Fritsche (2011) [108]	Cohort (all HG)	25	94	93	PPV 76%, NPV 99%
Youssef (2012) [109]	Cohort, reflex (negative cytology)	142	23	94	PPV 40%, NPV 88.5%
Kim (2014) [31]	Cohort (negative cystoscopy, suspicious cytology)	–	–	–	Association with Rec
Sideman (2015) [14]	Cohort, reflex (negative cystology and negative cystoscopy)	664	56	67	PPV 54.8%, NPV 67.9%
Lotan (2017) [7]	Marker-comparison prospective	157	33	–	NPV 92%

*PPV* positive predictive value, *NPV* negative predictive value, *Rec* recurrence

Despite this high variance in performance, several studies have suggested a clinical utility of FISH in specific clinical scenarios. Seideman et al. investigated the role of FISH in patients with previous BCa who presented at follow-up with both negative cytology and cystoscopy. In such cases, FISH was able to anticipate disease Rec at a median follow-up of 26 months; mean time to Rec was 12.6 months in patients who were FISH positive compared to 17.9 months in those with negative FISH (*p* = 0.03) [15].

Another scenario for its use is the presence of an unsuspicious cystoscopy and equivocal/atypical cytology or in patients with abnormal cystoscopy, in which the presence of cancer is not clear. In these patients, FISH could be used as a reflex test to adjudicate the significance of these findings [14]. Two prospective studies have evaluated the role of FISH in the setting of atypical cytology or cystoscopy [29]. In these studies, a positive FISH had a high PPV and led to the recommendation to consider biopsy, upper tract imaging or close cystoscopic re-evaluation. Schlomer et al. reported a sensitivity and NPV of 100% [30]. In fact, based on these and other studies, the guidelines specifically recommend the use of a urine marker in this setting [31].

In terms of the predictive role of FISH, Kim et al. [32] reported the predictive role of FISH in NMIBC patients with negative cystoscopy and suspicious cytology in which positive FISH was a significant predictor of Rec (HR 2.35; 95% CI 1.42–3.90, *p* = 0.001) in multivariable analysis and for Prog (HR 3.01; 95% CI, 1.10–8.21, *p* = 0.03) in univariable analysis. It has further been shown that the decision to omit bladder biopsy because of negative UroVysion in patients with atypical cytology and negative or equivocal cystoscopy was cost-effective and may allow reduction of unnecessary adverse events [33].

#### Phase II–III markers

##### Cxbladder monitor

Studies investigating the performance of this test are listed in Table 6. The Cxbladder Monitor test is based on the detection of four mRNAs that are significantly increased in the urine of BCa patients and of another mRNA that is associated with non-malignant conditions, included to reduce false-positive results. This marker panel was designed to optimize sensitivity. While this has improved test performance for this goal with reported sensitivity and NPV of 93 and 97%, respectively, it does come at the cost of a low specificity [34]. Sensitivity reached 95% in recurrent disease with high risk of Prog, and false-negative findings were reported in only 1.5% of cases. These data have been successfully externally validated [7]. This test may allow clinicians to postpone or avoid cystoscopy in patients under surveillance, who are at low risk of Rec. This might lower the cost and potential morbidity while improving quality of life in these patients.

**Table 6:** List of studies investigating the performance of CXbladder monitor and UBC tests in surveillance setting in patients with non-muscle invasive bladder cancer

Marker	Author (year) reference	Study design	No. of patients	Sensitivity (%)	Specificity (%)	Other end points
CXbladder monitor	Lotan (2017) [7]	Marker-comparison prospective	803	91	–	NPV 96%
	Kavalieris (2017) [33]	Cohort prospective	763	93	–	NPV 97%
Bladder cancer (UBC) test	Kibar (2006) [82]	Cohort/case–control	60/30	60	92.3	–
	Giannopoulos (2001) [79]	Marker-comparison prospective	95	80	–	–
	Babjuk (2008) [22]	Cohort prospective	88	12	97	–
	Pichler (2017) [19]	Marker-comparison prospective (UBC qualitative)	75	61.3	77.3	PPV 65.5%, NPV 73.9%
	Pichler (2017) [19]	Marker-comparison prospective (UBC quantitative)	75	64.5	81.8	PPV 71.4%, NPV 76.6%

*PPV* positive predictive value, *NPV* negative predictive value

##### Bladder cancer test (UBC test)

The Bladder Cancer Test (UBC) is available in two different assays. One is a quantitative, ELISA-based assay (UBC IRMA) while the other is a qualitative POC-based assay (UBC Rapid). Both of these detect the presence of cytokeratins 8 and 18 in the urine which play an active role in tumor invasion. Sensitivity and specificity rates vary from 12 to 80% and from 77 to 92%, respectively. When performed in combination with cytology, UBC has been reported to increase the overall sensitivity to 77.4% while decreasing its specificity [20]. Conversely to the other markers UBC is a point-of-care test, with results available within 10 min, and therefore, with possible and incontrovertible clinical advantages. However, the clinical utility of the UBC test in the follow-up of NMIBC patients remains unconvincing.

#### Phase I–II markers

##### XPERT BC monitor

The XPERT Bladder Cancer (BC) Monitor is an mRNA-based urinary marker test developed for BCa surveillance. It measures the levels of five different target mRNAs (ABL1, CRH, IGF2, UPK1B and ANXA10) by real-time PCR. These mRNAs are related to cell proliferation and survival, signal transduction and response to neuroendocrine stress. Advantages of the test are mainly related to its rapidity (< 2 min of hands-on sample preparation and total PCR time of around 90 min). The first results of XPERT BC test have been recently reported [35]. In this study, a total of 155 urine samples of 140 patients with history of NMIBC were collected during routine follow-up. The test showed to be significantly superior to urinary cytology in terms of overall sensitivity (84 vs 33%) and NPV (93 vs 76%), while overall specificity did not differ between tests (91 vs 94%). Moreover, while the sensitivity of the XPERT BC Monitor was 100% in patients with high-grade tumors, it remains relatively high (compared to cytology) also in patients with low-grade disease (77%). These findings are encouraging and call for prospective trials to go ahead in the test’s validation process.

##### Soluble FAS

Soluble FAS (sFAS) is an antiapoptotic protein released by BCa cells, protecting them from host antitumor activity. They are released in serum and urine, where they can be detected and measured by ELISA. Svatek et al. measured sFAS levels in 188 patients at risk for BCa Rec [36]. Higher levels of sFAS were associated with tumor stage ≥ *T*1, positive urinary cytology results and higher NMP22 levels. sFAS was more specific than NMP22 and was able to predict the presence of BCa in patients with negative cytology, suggesting that sFAS may be helpful as a complement to cytology. On multivariable analysis, sFAS levels predicted the presence of tumor (OR 3.1, CI 95% 1.6–5.9) and invasive stage (OR 3.7, CI 95% 1.3–10.9). These results were confirmed by Yang and colleagues who found that sFAS levels were an independent predictor of RFS (HR 1.4, 95% CI 1.1–1.9) [37]. However, the sFAS assay still needs to be refined and standardized prior to its introduction into clinical care. Moreover, further large, prospective, multicenter phase III trials are also needed.

##### Hyaluronic acid

Hyaluronic acid (HA) is a non-sulfated glycosaminoglycan that is involved in cell adhesion and proliferation, promotes tumor metastasis, and has been shown to be elevated in a variety of tumors. It is degraded by hyaluronidase enzyme (HAase) into small fragments that promote angiogenesis [16]. HA and HAase have been found to be elevated in the urine of BCa patients; they are measured with ELISA and RT-qPCR [38]. Lokeshwar et al. [25] performed the HA–HAase test on 225 urine samples from 70 BCa patients. Sensitivity, specificity, PPV and NPV were 91, 70, 92 and 67%, respectively. In this study, the HA–HAase test demonstrated superior performance compared to the BTA Stat test. However, to date, this is the only published study focusing on the role of the HA test in the follow-up of NMIBC patients.

##### Survivin

Survivin is an antiapoptotic protein that is almost exclusively expressed by malignant epithelium, which inhibits apoptosis, promotes cell proliferation, and induces/enhances angiogenesis. Shariat et al. [39] evaluated levels of survivin and NMP22 in voided urine samples from 117 BCa patients undergoing cystoscopy and 92 controls. Survivin had superior sensitivity (64%), specificity (93%), PPV (92%) and NPV (67%) compared to both NMP22 and urine cytology. Moreover, higher levels of survivin were associated with an increased risk of more advanced histologic grades [40]. While these findings are promising, this is the only published study evaluating the role of urinary survivin in the follow-up of NMIBC. Moreover, due to its low linearity, the assay remains experimental and requires further development and standardization.

##### Telomerase

Telomerase is a ribonucleoprotein that synthesizes telomeres at the ends of chromosomes, thus ensuring genome stability. Several tumors, including BCa, show telomerase hyperactivity that protects the chromosomes of cancer cells and thereby produces potential immortality [16]. One study explored the role of telomerase in patients with previous diagnosis of NMIBC [41]. Brems et al. measured levels of telomerase in 123 patients with BCa (12 incident cancers and 111 follow-up visits) and found that higher telomerase levels were associated with Rec (sensitivity and specificity of 62 and 84%, respectively), and that combining telomerase with cytology led to an increased sensitivity, but to a decreased specificity in diagnosis when compared to cytology alone. The lack of specificity of telomerase activity makes it a biomarker of little value with a high false-positive rate. Moreover, assays for telomerase still need to be standardized and validated.

##### Fibroblast growth factor receptor 3 (FGFR3) and methylation biomarkers

About 70% of low-grade NMIBC has been found to harbor a mutation in the fibroblast growth factor three (*FGFR3*) gene [42]. Since this mutation seems to be able to identify a subgroup of patients with good prognosis [43], its analysis could serve as biomarker not only for the detection of recurrence during follow-up of low-grade cancers, but also to identify patients with a low risk of progression. Zuiverloon et al. determined the *FGFR3* mutation status on 200 low-grade NMIBC patients [44]. The sensitivity of the assay for the detection of concomitant recurrence was 58%, while a *FGFR3*-positive test was associated with a 3.8-fold higher risk to have a recurrence during follow-up.

Several studies have explored the role of different DNA methylation genes as urinary biomarkers in the surveillance setting. Actually, changes in DNA methylation are stable, tend to occur early during carcinogenesis and can be detected in cells released in urine. Methylation biomarkers’ performance seems to be highly variable depending on the studies. Su et al. analyzed the DNA methylation levels of six markers in 368 urine samples from 90 patients with NMIBC and reported a sensitivity and specificity of 89 and 97%, respectively [45]. Similar findings were reported for other methylation markers [46, 47]. On the contrary, other studies did not confirm these promising findings: Abern et al. developed a PCR assay based on the methylation status of *TWIST1* and NID2. In a prospective surveillance trial, a relatively low sensitivity of 58% and specificity of 66% were reported [48]. Based on these results, methylation gene panels are, to date, far away to be implemented in routine clinical practice.

The combination of *FGFR3* status and methylation biomarkers has been tested with promising results initially. Beukers et al. investigated the role of *FGFR3* and of the methylation genes *TERT* and *OTX1* in 977 NMIBC patients [49]. The combination of the three assays detected 57% of Rec with a specificity of 59%. The diagnostic value of *FGFR3* was limited in HG tumors, reflecting the lower percentage of its mutation in this subgroup of patients. *FGFR3* mutation was found to predict Rec over time: false-positive urine samples from patients with *FGFR3* mutations were followed by a recurrence within 2 years in 73% of patients. Similarly, *FGFR3* mutation in combination with a 3-plex methylation assay was found to have a sensitivity of 79% and a specificity of 77% in detection of a tumor recurrence [50]. Finally, combining *FGFR3* mutational status with methylation measurement of a set of DNA methylation markers (*HS3ST2, SEPTIN9* and *SLIT2*) led to a sensitivity/specificity/NPV of 94.5/75.9/98.5%, respectively, for detection of a recurrent tumor [51]. Combined analyses of *FGFR3* mutation and DNA methylation markers could be valuable for risk stratification of patients with NMIBC and for surveillance setting, providing the basis for a promising non-invasive urinary test.

##### Angiogenesis markers

Vascular endothelial growth factor (VEGF) is associated with tumor angiogenesis and is secreted in urine by BCa cells. It is detectable and measurable with ELISA. High levels of urinary VEGF have been related to poor RFS in patients with previous NMIBC [52]. However, these preliminary data and the role of VEGF in a surveillance setting remain to be confirmed.

##### Others

DNA microsatellites are short tandem DNA repeats resulting from a failure of DNA mismatch repair and are, therefore, involved in tumor cell transformation. They have been studied as urinary markers [53]. However, the low sensitivity and specificity (58 and 73%, respectively) are not sufficient to recommend their implementation in clinical practice.

Finally, several other investigational biomarkers such as Ki-67, Fibronectin, CD44 antigen and BCa cell metabolites have been suggested to be related to the presence of BCa and have been studied in BCa detection [54]. However, their role in BCa surveillance remains uninvestigated or preliminary.

## Urinary biomarkers for the prediction of BCG response

### Performance criteria desired

The goal of urine markers in this specific scenario is to predict whether BCG treatment will be effective and to monitor its effectiveness.

### Urinary markers rated according to quality of research—according to a phased approach

BCG efficacy appears to depend on the ability to generate an appropriate immune response, including mainly CD4 + T cells and CD8 + cytotoxic T lymphocytes (CTLs) [55]. The therapeutic antitumor effect of intravesical BCG involves the interactive activity of urothelial cells (including BCa cells) and cells of the immune system. BCa cells are able to internalize BCG through phagocytosis and macropinocytosis. After this event, BCa cells directly secrete immune-activating effectors such as chemokines and cytokines (i.e., IL-6, IL-8, GM-CSF and TNF). Moreover, BCa cells are functioning antigen-presenting cells, presenting BCG antigens to CD4 + T cells. BCa cells are finally killed by cytotoxic immune cells, secretion of soluble factors and the direct action of BCG [56].

#### Phase III–IV markers

According to AUA/SUO Guidelines, “clinicians may use biomarkers to assess the response to intravesical BCG (UroVysion^®^ FISH) and adjudicate equivocal cytology (UroVysion^®^ FISH and ImmunoCyt®)” [14]. UroVysion, performed 6 weeks after the last BCG instillation, was reported to predict BCG failure (either disease persistence or Rec), both in patients with positive urine cytology and in those with non-definitive cytology [57]. Abnormal results obtained from serial measurements of UroVysion at baseline (before BCG), at 6 weeks (before the last BCG induction cycle) and at 3 months (before the first surveillance cystoscopy) were significantly associated with Rec and Prog. The term “molecular BCG failure” was proposed to define those patients with abnormal FISH results and negative first cystoscopy [58]. However, this concept remains to be validated.

#### Phase II–III markers

The presence and levels of BCG-induced cytokines in urine can be measured and might predict the clinical response to BCG. Kamat et al. [59] measured levels of 12 urinary cytokines and calculated changes from baseline during BCG treatment, developing a nomogram, based on nine inducible cytokines [IL-2, IL-8, IL-6, IL-1ra, IL-10, IL-12(p70), IL-12(p40), TRAIL, and TNF-α] that could predict Rec with an accuracy of 85.5% (95% CI 77.9–93.1). Previously, several studies had focused on the role of urinary IL-2 levels in patients treated with BCG [60–62]. High levels of IL-2 in urine after BCG administration were associated with increased risk of Rec. Similarly, elevated urinary levels of IL-8 and IL-18 after BCG therapy predicted RFS [63]. At a cut-off threshold of 112 pg/mL, IL-8 measured 2 h after BCG instillation predicted Rec with a sensitivity of 53%, specificity of 89%, PPV of 73%, and NPV of 77% [64]. Finally, the IL-6/IL-10 ratio was able to predict BCG response and RFS in patients at intermediate risk with a sensitivity and specificity of 83 and 76%, respectively [65].

The suggested role of biomarkers involved in the prediction of BCG response is summarized in Table 7.

**Table 7:** List of biomarkers predicting response to intravesical BCG in patients with non-muscle invasive bladder cancer

Urinary biomarker	Patients	Outcome investigated	Magnitude of effect	Ref.
IL-2	CIS	RFS	HR 0.368; 95% CI, 0.029–0.895	[60]
IL-6/IL-10	High-risk NMIBC	RFS	HR 4.09; 95% CI 2.59–6.28	[64]
IL-8	Primary NMIBC	Recurrence	Sensitivity 53.3%, specificity 88.5%, PPV 72.7%, NPV 76.7%	[63]
IL-18	NMIBC	Recurrence	Different IL-18 level’s statistical significance in patients with or without recurrence	[62]
Nomogram [IL-2, IL-8, IL-6, IL-1ra, IL-10, IL-12(p70), IL-12(p40), TRAIL, and TNF-α]	Intermediate- and high-risk NMIBC	Recurrence	Accuracy 85.5%; 95% CI 77.9–93.1%	[58]
UroVysion	NMIBC	Persistence or recurrence	HR 5.6; 95% CI, 2.5–12.2	[56]

*HR* hazard ratio, *CI* confidence interval, *PPV* positive predictive value, *NPV* negative predictive value, *CIS* carcinoma in situ, *NMIBC* non-muscle invasive bladder cancer

## Conclusions

Many urinary biomarkers have been evaluated to predict Rec and/or Prog in the follow-up of NMIBC patients. Current commercially available urinary biomarker tests provide inadequate performance and are neither routinely used nor recommended in clinical practice. However, some of them could be useful in specific clinical scenarios such as a negative cystoscopy and inconclusive/suspicious urinary cytology. Moreover, preliminary results have shown that urinary markers such as FISH might be useful to predict BCG response. Despite this, prospective well-controlled trials are lacking and are needed to provide high-evidence data before these tests can be integrated into clinical use.

Several new biomarkers have recently been developed and are currently under investigation. Despite encouraging results, their clinical relevance remains to be established. Most of them have not yet gone through all of the necessary phases of marker development and the testing process. However, a renewed interest about biomarkers is growing and it is probable that in the next few years, a generation of new biomarkers will enter into the clinical setting.
